# ER Stress and Iron Homeostasis: A New Frontier for the UPR

**DOI:** 10.1155/2011/896474

**Published:** 2010-12-20

**Authors:** Susana J. Oliveira, Maria de Sousa, Jorge P. Pinto

**Affiliations:** ^1^Instituto de Biologia Molecular e Celular, Universidade do Porto, Rua do Campo Alegre 823, 4150-180 Porto, Portugal; ^2^Instituto de Ciências Biomédicas Abel Salazar, Largo Prof. Abel Salazar 2, 4099-003 Porto, Portugal

## Abstract

The C282Y mutation of HFE accounts for the majority of cases of the iron overload disease Hereditary Hemochromatosis (HH). The conformational changes introduced by this mutation impair the HFE association with *β*_2_-microglobulin (*β*_2_m) and the cell surface expression of the protein: with two major consequences. From a functional perspective, the ability of HFE to bind to transferrin receptors 1 and 2 is lost in the C282Y mutant, thus affecting hepcidin regulation. Also due to the faulty assembly with *β*_2_m, HFE-C282Y molecules remain in the endoplasmic reticulum (ER) as aggregates that undergo proteasomal degradation and activate an Unfolded Protein Response (UPR). UPR activation, regardless of the ER stress stimuli, was shown to reshape the expression profile of iron-related genes and to decrease MHC-I cell surface expression. The possibility of a HFE-C282Y-mediated interplay between the UPR and iron homeostasis influencing disease progression and the clinical heterogeneity among C282Y carriers is discussed. The responsiveness of the ER chaperone calreticulin to both ER and iron-induced oxidative stresses, and its correlation with HH patients' phenotype, reinforce the interest of dissecting the UPR signaling/iron metabolism crosstalk and points to the potential clinical value of use of pharmacological chaperones in HFE-HH.

## 1. Introduction

Occupying a central position in the secretory route, the endoplasmic reticulum (ER) performs a vast array of functions that includes the biosynthesis, folding, assembly, and posttranslational modification of secretory and membrane-targeted proteins [1]. The accuracy of this variety of processes relies on specialized luminal conditions, thoroughly maintained by stringent quality control mechanisms [2]. Despite the sophistication of such mechanisms, certain physiological states and exogenous stimuli can compromise this optimal folding environment and are generally referred to as ER stress [3]. 

The realization that there is a functional intersection between ER stress and iron metabolism emerged first from studies of the genetic disorder of iron overload, Hereditary Hemochromatosis (HH) type 1. Arising from a defective regulation of iron absorption, HH is intrinsically related to the gene *HFE* [4]. The product of this gene shares structural homology to a major histocompatibility complex class I (MHC-I) protein, requiring association with *β*_2_-microglobulin (*β*_2_m) for cell surface expression [5, 6]. In contrast to conventional MHC-I molecules, HFE is unable to bind peptides and has not been implicated in antigen presentation functions [6]. Instead, the HFE-*β*_2_m heterodimer was shown to bind transferrin receptor (TfR)1, thus competing for its interaction with diferric transferrin [7, 8]. The binding capacity of HFE was later extended to TfR2 [9]. C282Y and H63D are the two point mutations of HFE commonly underlying HH [5]. The former, resulting from a G-A transition that replaces the amino acid cysteine by tyrosine at position 282, is carried by the majority of HH patients (>85%), while the H63D, consisting in a C-G transition that determines a histidine to aspartate substitution at position 63, acquires clinical significance in C282Y/H63D compound heterozygotes [10, 11].

The surprise caused by the discovery of a MHC-I-like protein partaking in iron homeostasis was diluted by previous research, fertile in reports describing immunological abnormalities in HH patients. Accordingly, higher CD4^+^/CD8^+^ ratios [12], later attributed to defective numbers of CD8^+^ T cells [13], were consistently found in HH subjects in comparison to control individuals. Complementing these data, increased clinical severity of the disease was observed in the context of a lower CD8^+^ T lymphocyte pool [14]. Discrepancies at the functional level were likewise revealed, with CD8^+^ T cells from HH patients exhibiting impaired cytotoxic and CD8-p56lck activities when compared to healthy controls [15, 16]. A glimpse for the structural homology with MHC-I molecules was also provided by the spontaneous iron overload phenotype developed in *β*_2_m^−/−^ mice [17].

The elucidation of the genetic background of HH, in concert with the rapid adoption of *HFE* mutation testing in clinical practice, soon revealed a poor penetrance of the disease in C282Y carriers [18]. A remarkable phenotypic heterogeneity among patients was noticed as well [19], suggesting the existence of additional factors modifying this genetically determined disorder. Modifiers of the clinical and immunological traits of HH have been actively pursued, namely, genetic ones [20]. In light of the recent findings, the Unfolded Protein Response (UPR) has emerged as a promising candidate. 

## 2. The C282Y Mutation and the UPR

By blocking the formation of a disulphide bond in the *α*_3_ domain of HFE, the C282Y mutation prevents the assembly with *β*_2_m [10, 21]. As a consequence, the mutant protein fails to progress through the secretory pathway and remains in the ER as high molecular weight aggregates [10, 22, 23] that undergo proteasome-dependent degradation [10]. Besides the presence of such intracellular aggregates, cells expressing the C282Y mutant version of HFE also display decreased surface expression of MHC-I molecules [24]. Seeking the molecular details underlying this observation and inspired by the ER retention of the C282Y mutant, de Almeida and coworkers established the UPR as a mechanism mediating the HFE/MHC-I crosstalk [25]. Both the UPR/MHC-I interplay and the ability of the C282Y faulty HFE to elicit UPR activation were later independently confirmed [26, 27]. 

UPR is the designation of the specialized signaling circuits developed by cells to counteract the luminal accumulation of misfolded client proteins and rebalance the load/capacity ratio of the ER threatened by physiological states and exogenous conditions as diverse as potent secretory activity, disruption of Ca^2+^ stores, alteration of redox status, energy/nutrient deprivation, expression of mutant substrates, and viral infection [28, 29]. Tailored to restore ER homeostasis, the UPR combines multiple synergistic strategies that encompass global suppression of protein synthesis and translocation into the ER, transcriptional induction of ER chaperones and foldases to face the increased folding demands, and improvement of the ER-associated degradation (ERAD) machinery to bolster the clearance of irreparably unfolded proteins [3, 30]. If the prosurvival attempts are exhausted and ER damage prevails, UPR-induced proapoptotic programs are executed [31]. 

In mammalian cells, three ER-resident transmembrane proteins operate as proximal sensors and define the major UPR signaling pathways: double-stranded RNA-dependent protein kinase-like ER kinase (PERK), inositol-requiring enzyme (IRE)1, and activating transcription factor (ATF)6 [3, 30]. Notwithstanding this diversity, association with the ER-resident chaperone immunoglobulin heavy chain-binding protein (BiP) is proposed as a common regulator of the ER transducers. Under unstressed conditions, BiP binds the luminal domains of all sensors, rendering them inactive. As unfolded proteins congest the ER, BiP is competitively titrated away, allowing the PERK-, IRE1-, and ATF6-dependent cascades to proceed [32, 33]. Alternative models of sensing have been presented as well. It was suggested, for example, that the MHC-like groove displayed by yeast IRE1 directly detects and binds misfolded clients in the ER lumen, which would contribute to IRE1 activation [34]. The groove dimensions in the related human protein, too narrow to accommodate peptides, have weakened the likelihood of this mechanism, however [35]. The two perspectives were recently reconciled by a model postulating that direct interaction with unfolded proteins is required for IRE1 activation, whereas BiP serves both as buffer and timer of UPR activity [36]. 

Encouraged by the misfolding nature of the C282Y HFE protein and the subsequent UPR activation, categorization of HH as a conformational disorder has been claimed [37, 38]. A reasonable explanation for the aforementioned genotype-phenotype inconsistencies amongst HH patients could therefore rely, at least in part, on dissimilar individual abilities to mount an appropriate protective response towards the C282Y mutant client. Albeit attractive, this hypothesis is far from consensual. One argument militating against it relates with the low tissue levels of HFE expression, recently estimated below 0.53 nmol/g of total protein in human liver [39]. Nonetheless, and despite some controversial data [40], increased hepatic mRNA expression of HFE was reported in iron-supplemented mice [41, 42], a trend also recapitulated by microglia-derived cells subjected to stressor agents and serum deprivation [43]. Accordingly, one could envisage a scenario in which the basally innocuous HFE pool may accumulate to levels that clog the ER with the C282Y misfolded variant as iron overload progresses in HH, thus favoring ER stress conditions. Whether and how this vicious cycle-based model influences the *in vivo* pathophysiology of HFE-linked HH definitely deserves assessment. 

## 3. The UPR Crosstalk with Iron Metabolism

### 3.1. Expression Profile of Iron-Related Genes

Originally described as a check-and-balance program focused on the recovery of stress-corrupted ER folding homeostasis, the boundaries of UPR have been steadily broadened to cell differentiation, metabolic and inflammatory processes [44–46]. Suggestive evidence for an intersection with iron metabolism was first provided fortuitously by differential gene expression screenings. Two such examples are the increased transcript levels of the ER chaperones calreticulin (CRT) and BiP found in iron-burdened astrocytoma cells [47] and the transferrin gene downmodulation reported in stable transfectants of the stress-inducible transcription factor CCAAT/enhancer-binding protein (C/EBP) homologous protein (CHOP) [48]. Likewise, proteomic analysis revealed increased hepatic BiP expression in dietary iron-loaded mice [49], although *in vitro* studies using the human hepatoma HepG2 cell line have failed to confirm this result (Pinto et al., unpublished data). Despite all these cues, the interplay of UPR and iron homeostasis has remained a hitherto unexplored field.

By exposing a hepatocyte-derived cell line to chemical agents impairing disulphide bond formation (dithiothreitol and homocysteine) in ER client proteins, we recently demonstrated that ongoing ER stress significantly reshapes the expression profile of iron-related genes, namely, hepcidin, ferroportin, and ferritin H. Using this experimental model, the molecular mechanism behind the biphasic modulation of hepcidin was also deciphered, with the nuclear factors C/EBP*α* and CHOP being implicated [50]. The interplay between iron metabolism and the UPR signaling pathways was independently corroborated by Vecchi et al. that, after stressing hepatoma-derived cells with the ER-to-Golgi transport inhibitor brefeldin A, calcium ionophore A23187 and tunicamycin, reported increased levels of hepcidin transcripts, a pattern also detected in the liver of tunicamycin-treated mice. The stimulation of hepcidin was attributed to cAMP response element-binding protein H (CREBH)-dependent activation of its promoter [51]. The mechanisms proposed by the two studies [50, 51] for hepcidin induction under stress scenarios are not incompatible and could conceivably coexist.

### 3.2. Calreticulin, a Chaperone Related to Clinical Expression of Iron Overload

As discussed earlier, UPR activation is characterized by the induction of several ER stress-responsive proteins. One of these molecules is CRT, a 46 kDa ER-resident lectin chaperone with Ca^2+^-buffering capacity, specifically devoted to the folding of a large fraction of clients traversing the secretory route, glycoproteins. 

In addition to the chaperone function, CRT is involved in protection from stress usually not included in the UPR-related ER stress category. CRT synthesis is induced by heat shock [52–54] and heavy metals [53], which has recently drawn attention to the role of this protein in protection from oxidative stress [55]. CRT, together with the other ER chaperone BiP, protected renal epithelial cells against lipid peroxidation induced by the prooxidant reagent tert-butyl hydroperoxide [56]. Production of the antioxidant nitric oxide leads to overexpression of CRT in pancreatic beta cells [57]. Also, hypoxic conditions, such as those found in wounds, can lead to overexpression of CRT by 2 to 3 folds, with a 7-fold increase observed in response to H_2_O_2_-induced oxidative stress during rat cardiomyocyte injury [58]. 

**Figure 1:**
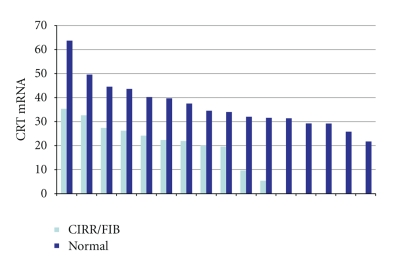
CRT expression and HH-related symptoms. *CRT* mRNA expression in PBMCs from HFE C282Y homozygous patients. Each bar represents an individual subject, ordered by CRT level and divided according to the presence (light blue bars) or absence (dark blue bars) of cirrhosis/fibrosis (CIRR/FIB), modified by Porto from original data from Pinto et al., 2008 [59].

A possible protective role for CRT was found in C282Y homozygous HH patients, in which the iron-induced oxidative stress and an UPR triggered by the unfolded C282Y-mutated HFE protein coexists. In this study [59], a negative association was found between the levels of CRT in peripheral blood mononuclear cells (PBMCs) and the severity of HH clinical manifestations (Figure 1). Although the mechanism underlying the variability in CRT expression among HH patients has not been elucidated, the finding of a positive association between *CRT* mRNA levels, BiP expression, and numbers of monocytes (the PBMC population with the highest HFE expression) favors the interpretation that CRT expression is being modulated by the UPR in PBMCs from C282Y^+/+^. 

The involvement of CRT in cellular protection from oxidative stress is possibly the mechanism underlying the changes in CRT expression in response to increased intracellular iron levels observed in colorectal adenocarcinoma (Caco-2) and hepatocarcinoma (HepG2) cell lines [59, 60]. In the Núñez et al. study [60], the iron-induced increase in CRT expression was effectively blocked by the antioxidant quercetin, whereas Pinto et al. showed, using both over-expression and siRNA-mediated silencing, that CRT upregulation is necessary to prevent iron-induced ROS accumulation [59].

**Figure 2:**
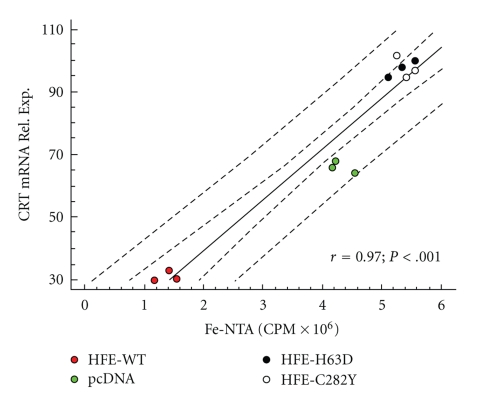
Regulation of CRT mRNA by iron. Linear regression analysis of the correlation between intracellular iron levels (3 *μ*M ferric nitrilotriacetate (^55^Fe–NTA) were used as iron source), and* CRT* mRNA pools in HepG2 cells overexpressing the WT or mutated (C282Y or H63D) HFE variants (HepG2 cells transfected with the empty pcDNA3 vector were used as negative control). Each dot represents the average of one experiment with three replicates. *r* = 0.97; *P* < .001. Inner dashed curves represent 95% confidence intervals for the mean value of CRT at any selected CPM value. Outer dashed lines are 95% prediction limits for new observations. Modified from Pinto et al., 2008 [59].

How could CRT exert its role in the protection of the cell against iron-induced oxidative stress? An answer might be given by mobilferrin, a cytosolic protein involved in the intracellular transport of iron during intestinal iron absorption [61]. Rat mobilferrin and CRT share 100% homology in the amino-terminal amino acid sequence; both proteins have the same apparent molecular weight and isoelectric point, and the antibodies raised against one of the proteins cross-react with the other [62]. Mobilferrin is thus considered to be an isoform of CRT, although further studies are needed to confirm this hypothesis. The increase in CRT/mobilferrin in a situation of augmented intracellular iron levels (Figure 2) could be a response of the cell to the need for more iron-binding capacity, to ameliorate the iron-induced oxidative stress. In view of the presence of CRT in several intracellular (and extracellular) locations, this role could take place in a variety of compartments, where the need for iron buffering would be present. One such compartment is the nucleus, where the presence of hydroxyl radicals generates a whole series of DNA damage, namely, single- or double-strand breaks, abasic sites and base and sugar lesions [63, 64]. Interestingly, over the last two decades, several studies have reported the presence of ferritin, the main iron-storage protein, in cell nuclei [65]. The earliest observations of nuclear ferritin were made in mice hepatocytes following iron overload [66]. Later, the presence of ferritin in the nucleus was found to correlate with higher resistance to UV and H_2_O_2_-induced DNA damage on corneal epithelial cells [67, 68], suggesting the need for protection from iron-induced oxidative stress in the nucleus.

 CRT participation in the response to oxidative stress may be a component of the broader involvement of the ER in cellular protection from a varied category of insults, namely those involving the iron overload toxicity. The predominant location of CRT and other ER proteins within the hyperoxic environment of the ER increases their susceptibility to oxidative damage [69], which, along with posttranslational modifications, can affect both the function and cellular location of these proteins [70]. All concurrent hypotheses agree that oxidative stress-induced ER stress leads to extracellular release of CRT and other ER proteins. The mechanisms involved in the translocation of the protein to the extracellular compartment are not yet fully understood, although they seem to involve triggering of apoptotic cell regulatory proteins (caspases, Bap31, Bax activation), an ER stress response leading to the phosphorylation of the eukaryotic initiation factor eIF2*α* and active exocytosis [71–74].

### 3.3. Calreticulin, at the Crossroads of MHC-I Expression and Iron Overload

Impaired cell surface expression of MHC-I molecules is another feature found in C282Y^+/+^ hemochromatosis patients besides iron overload. In view of the UPR/MHC-I crosstalk revealed in recent years [25, 26], together with the iron burden developed by *β*_2_m and MHC-I KO murine models [17, 75], the chaperone activity of CRT might gain a renewed relevance in the context of MHC-I assembly. The process takes place in the ER with the assistance of a number of chaperones and folding factors, which include CRT. The complex to be assembled consists of a glycosylated heavy chain, a *β*_2_m molecule, and a peptide. Peptide loading of MHC-I molecules is the final step of an intricate pathway that results from the adaptation of a quality control cycle that regulates the folding of conventional glycoproteins [76]. The complex responsible for peptide loading comprises the peptide transporter TAP (transporter associated with antigen processing), ERp57, CRT, calnexin (CANX), and tapasin (TAPBP, TAP-binding protein) [77, 78]. Both CRT and CANX promote the assembly of MHC-I and retain incompletely assembled complexes in the ER [78]. CRT binds to a monoglucosylated N-linked glycan at Asn86 of the MHC-I heavy chain with a dissociation constant of approximately 1 *μ*M [79], following CANX release. 

The importance of CRT in MHC-I processing is illustrated by the suboptimal MHC-I assembly in CRT KO cells [80], with MHC-I expression and stability at the cell surface being rescued by the reintroduction of a lectin-deficient CRT mutant [81]. Although the mechanisms underlying the involvement of the MHC-I in iron metabolism remain unclear, the CRT role in MHC-I assembly/expression may, at least in part, explain the protective function of this protein in iron overload [59].

## 4. The UPR Crosstalk with Iron Metabolism: Putative Physiological Significance

The central regulator of iron homeostasis is hepcidin, a 25-residue peptide hormone. Mainly secreted by hepatocytes [82], hepcidin binds to the membrane iron exporter ferroportin, triggering its internalization and lysosomal degradation [83]. Iron egress from enterocytes and macrophages is therefore inhibited, ultimately restricting the availability of the biometal in circulation. The pleiotropic nature of hepcidin arises from its responsiveness to iron, inflammation, anemia, and hypoxia [84, 85]. Hepcidin is physiologically stimulated by increased iron stores and inflammation, with the converse occurring in the remaining conditions. This versatility is mirrored by the array of signaling pathways coordinating hepcidin transcription as yet identified [86–91], now extended to the ER stress chain of events. 

A systemic impact of the UPR has been disclosed through the connection to insulin secretion and peripheral resistance [92, 93], glucose homeostasis [94], and inflammation [95]. With the UPR-induced hepcidin modulation [50, 51], a new item can be coupled to this picture. By limiting duodenal iron absorption, hepcidin upregulation in this context may be part of a protective “strategy” to evade extra sources of stress, as those associated with iron-generated ROS. In line with hepcidin's antimicrobial role [82], consequences on the innate immunity are expected as well, thereby furthering the scope of the recently uncovered ER stress-mediated inflammatory responses [46, 96].

Besides the systemic impact underlined by hepcidin, repercussions of UPR activation are also manifested at the cellular iron metabolism level, as suggested by the modulation of ferroportin and ferritin H expression imposed by ER insults [50]. The mRNA enrichment of both genes in cells enduring ER stress may reflect an attempt to circumvent intracellular deposition of free iron either via its sequestration or export, respectively.

## 5. The UPR Crosstalk with Iron Metabolism: A Link to Pathological Conditions

Building evidence has coupled the ER stress response circuitries to multiple pathologies, namely diabetes, obesity, and neurodegeneration [97–99]. Apart from the above-described connection with HFE-linked HH, the novel association between ER stress and iron homeostasis may prove useful in furthering the understanding of neurodegenerative processes. In fact, iron accumulation in affected brain regions is a commonality of various neuropathologies, including Alzheimer's disease (AD) and Parkinson's disease (PD) [100]. Regardless of the yet uncertain mechanisms driving such deposition, the significance of inherent oxidative stress to neuronal damage has been increasingly recognized [101]. Belonging to the class of conformational disorders, protein misfolding and aggregation are also hallmarks of both AD and PD, probably potentiating neuronal cell death [99]. The neurodegeneration field may be therefore worth exploring for the dialogue between iron homeostasis and ER stress. One can conceive, for example, that the transcriptional reshaping triggered by UPR activation takes part on the brain iron imbalance observed in AD and PD. 

Another foreseeable repercussion of these new findings touches on the virus-iron metabolism-UPR defined triad. The ability of viruses to coopt the biochemical machineries of host cells to mass replicate themselves is a longstanding concept. One of the widely studied processes is the viral interference with multiple steps of MHC-class I antigen presentation route, likely evolved to elude immune surveillance [102]. Because iron availability is critical for efficient proliferation, an additional subversive approach triggered by viruses includes manipulation of host iron status. Despite our still tangential understanding of this strategy, progress has been made by demonstrating that TfR1 might be engaged in the viral entry process [103, 104]. Furthermore, US2 and Nef proteins encoded in the genomes of human cytomegalovirus (HCMV) and human immunodeficiency virus (HIV)-1, respectively, were shown to down regulate the cell surface expression of HFE [105, 106], presumably with the consequence of replenishing intracellular iron stores and benefit viral growth. Also supporting this interaction, repressed hepcidin synthesis was attributed to hepatitis C virus (HCV) infection [107]. The UPR, whose activation has been proven in infected cells [108–110], emerges as a plausible common denominator of the aforementioned viral strategies. In fact, by exploiting the UPR pathways, viruses might simultaneously: (i) guarantee ER expansion to accommodate massive production of viral proteins, (ii) impair MHC-class I presentation [25, 26], thus helping in the immune evasion endeavor, and (iii) tune the activity of host proteins involved in iron metabolism to ensure adequate supply of this biometal. 

The biological relevance of the UPR-induced modulation of iron homeostasis in the context of neurodegeneration and viral infection must be thoroughly characterized, warranting promising research directions.

## 6. HH: A Candidate for Pharmacological Chaperone Therapy?

In light of the current paradigm, the iron-dependent tuning of hepcidin is governed by the HFE/TfR1/TfR2 partnership [88]. Due to compromised cell surface expression, the C282Y variant of HFE fails to bind TfR1 or to stabilize TfR2 according to the prevailing transferrin saturation levels. Impairment of the adequate adjustment of hepcidin levels causes parenchymal iron deposition, with associated complications including cirrhosis, heart failure, diabetes, and arthropathy [4]. This loss-of-function model, however, challenges the identification of novel regulatory mechanisms mediated by the C282Y mutation, as those involving its ER retention. In fact, to what extent misfolding events triggered by the C282Y mutant influence the course of HFE-linked HH remains elusive. 

The standard therapy for HH consists in periodic blood withdrawals—phlebotomy—aimed at depleting excessive iron stores [4]. Early initiation of treatment efficiently prevents organ failure due to iron toxicity and restores normal lifespan, although arthropathy barely improves [111]. Nevertheless, the immunological anomalies reported in HH patients are not solved by the blood-letting therapy [16], supporting the existence of factors beyond iron overload dictating such traits. The protective role against oxidative stress recently attributed to CRT in HFE C282Y stable transfectants, along with the negative correlation between expression of this ER chaperone and the number of clinical manifestations presented by HH subjects [59], conveys the rationale for considering that pharmacological chaperones might be useful in the context of HFE-hemochromatosis.

The pharmacological manipulation of ER folding capacity and quality control systems has merited particular attention in recent years as promising therapeutic strategy for conformational disorders [112]. Relying on low-molecular-weight compounds that stabilize native conformations and compensate intrinsic folding deficits, this approach may limit aggregation episodes and/or rescue protein function [113–116]. The endogenous bile acid taurine-conjugated ursodeoxycholic acid (TUDCA) and the short-chain fatty acid derivate sodium 4-phenylbutyrate (4PBA) are two of such agents whose value in inhibiting the stress elicited by the HFE C282Y mutant was already established *in vitro* [23]. The former was shown to increase the stability of the C282Y protein, whereas reduced levels of intracellular aggregates were achieved by 4PBA supplementation. These results, underscored by the recently uncovered impact of ER stress in iron metabolism, may inspire the design of new approaches for treating HFE-HH, complementing the current clinical protocols. In this case, however, a thoroughly balanced chaperone therapy would be required in order to preserve the beneficial effects rendered by UPR activation, as those mediated by CRT against iron-generated oxidative stress [59].

## 7. Concluding Remarks

**Figure 3:**
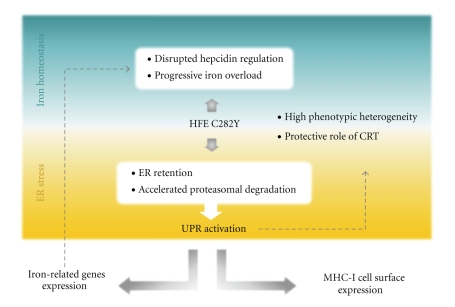
ER stressing iron homeostasis. *Iron homeostasis* (upper panel). Hepcidin is today thought to be the key regulator of iron homeostasis. Lower hepcidin levels have been seen associated with progression of iron overload in Hereditary Hemochromatosis (HH). HH is intrinsically related to the gene HFE and to a point mutation commonly known as C282Y replacing cysteine by tyrosine at position 282. A high proportion of HH patients are homozygous for the C282Y mutation. *ER stress* (lower panel). Studies by two separate groups showed in recent years that the C282Y mutation in HFE provoked the activation of the Unfolded Protein Response (UPR) leading the two groups to consider HH a conformational disorder. In addition, some clinical phenotypic heterogeneity reported in HH has been related to variation in levels of mRNA expression of the endoplasmic reticulum (ER)-chaperone calreticulin (CRT). UPR activation has also been shown by separate groups to affect hepcidin gene expression. The intriguing effect of the UPR on cell surface expression of MHC class I may also contribute to the phenotypic heterogeneity of HH through its putative peripheral effect on numbers of circulating CD8^+^ T cells.

Largely propelled by its implication in varied pathological conditions, interest in understanding the interface between ER stress and other cellular signaling pathways is growing considerably. Over the last decades substantial effort to elucidate the molecular components and mechanistic details of the UPR canonical cascades was set in motion, with remarkable insights being achieved including considering a role for chaperones in therapy. Our work establishing a connection between the UPR and the expression of genes relevant for the regulation of iron metabolism reviewed in the present paper extends this networking model, highlighting further the multitasking nature of the UPR. In addition, our studies of CRT mRNA expression in genetic iron overload revealed CRT as a possible significant chaperone at the crossroads of UPR and iron homeostasis with a possible protective effect towards the iron-mediated disease due to its antioxidative properties (Figure 3).

 Altogether, the title chosen, *ER stress and iron homeostasis: a new frontier for the UPR*, stresses with a few words the opening of a new promising avenue in UPR and chaperone research. It is hoped that with time facts will match the promise of words.
